# Correction: Chua et al. Cost-Effectiveness Analysis of HPV Extended versus Partial Genotyping for Cervical Cancer Screening in Singapore. *Cancers* 2023, *15*, 1812

**DOI:** 10.3390/cancers15184658

**Published:** 2023-09-21

**Authors:** Brandon Chua, Li Min Lim, Joseph Soon Yau Ng, Yan Ma, Hwee Lin Wee, J. Jaime Caro

**Affiliations:** 1 Saw Swee Hock School of Public Health, National University of Singapore, 12 Science Drive 2, Singapore 117549, Singapore; 2 Health Economics and Outcomes Research, Becton Dickinson Holdings Pte. Ltd., 2 International Business Park Road, The Strategy #08-08, Singapore 609930, Singapore; 3 Division of Gynaecologic Oncology, Department of Obstetrics and Gynecology, National University Hospital, 5 Lower Kent Ridge Rd., Singapore 119074, Singapore; 4 Department of Pharmacy, National University of Singapore, 18 Science Drive 4, Singapore 117543, Singapore; 5 School of Global and Population Health, McGill University, Suite 1200, 2001 McGill College Avenue, Montréal, QC H3A 1G1, Canada; 6 Department of Health Policy, London School of Economics and Political Science, Houghton Street, London WC2A 2AE, UK; 7 Evidera, 500 Totten Pond Rd., Waltham, MA 02451, USA

The authors wish to revise two words in Table 1 row 3, and the first paragraph of Section 2.3 of this article [1] that were overlooked in the final proofreading.

Table 1 row 3: “follow-up adherence” should be read as “follow-up non-adherence”; Section 2.3: the gross domestic product per capita in Singapore in 2021 should read “SGD 97,798” instead of “USD 97,798”.

This is the corrected paragraph:

**Table 1:** Inputs for the model.

Input	Base Case	Lower Limit	Upper Limit	Distribution	Reference
Number eligible	1,037,598	**-**	**-**	-	[27,28]
Screening coverage	48.2%	45.8%	50.7%	Beta	[30]
Follow-up non-adherence *	25.0%	0%	40%	-	[28] ^†^
**Clinical inputs**					
hrHPV					
Prevalence	9.2%	7.9%	10.5%	Beta	[28]
% non-HPV16/18	80.8%	70.3%	83.0%	Beta	[28]
% Group B	56.6%	51.0%	61.0%	Beta	[41]
% NILM	56.1%	-	-	-	[42]
ASCUS among:					
Group B	31.8%	-	-	-	[42]
Group A	40.6%	-	-	-	[42]
CIN1 regressing in 1 year	60.0%	45.0%	73.0%	Beta	[43]
Cancers among:					
CIN2+ diagnosis	2.6%	2.3%	2.9%	Beta	[44]
CIN2+ of Group B with ASCUS	2.6%	0.0%	10.0%	-	[44,45,46,47]
CIN2+ risk with:					
Group B with ASCUS	6.1%	2.6%	9.5%	Beta	[45,46,47,48]
Group A with ASCUS	14.2%	-	-	-	[48]
HPV16/18	21.9%	-	-	-	[48]
Non-HPV16/18 with LSIL+	16.4%	-	-	-	[48]
PSGI at repeat screening	57.1%	54.2%	60.1%	Beta	[49,50,51]
hrHPV 1 yr persistence	43.3%	41.8%	44.8%	Beta	[52]
HSIL/ASC-H 1 year post CIN1/negative for CIN	6.7%	5.7%	7.7%	Beta	[53]
ASCUS+/HPV+ 2 years post CIN1/negative for CIN	15.4%	13.8%	17.1%	Beta	[28]
Proportion stage I cancer	40.8%	-	-	-	[54]
Proportion stage II cancer	24.4%	-	-	-	[54]
Proportion stage III cancer	18.1%	-	-	-	[54]
Proportion stage IV cancer	16.7%	-	-	-	[54]
10-year cancer survival	45.4%	-	-	-	[40]
XGT repeat screenings	2	1	5	-	^†^
Annualized CIN2+ risk for					
HPV genotype persistence					
Same	5.7%	-	-	-	[55]
Change	1.9%	-	-	-	[55]
Regardless of genotype	3.3%	-	-	-	[55]
Multiplier for CIN2+ risk	1	0.7	1.38	Normal	^†^
Annualized CIN2+ risk for CIN1/negative for CIN					
1 negative pap smear	1.1%	-	-	-	[56]
ASCUS/LSIL upon follow-up	2.1%	-	-	-	[56]
ASC-H upon follow-up	5.3%	-	-	-	[56]
HSIL+ upon follow-up	3.4%	-	-	-	[56]
**Cost inputs SGD (USD)**					
Clinic visit	75 (89)	37 (44)	113 (134)	Normal	[34]
Cytology	79 (94)	39 (46)	119 (141)	Normal	[34]
HPV DNA (PGT)	115 (137)	57 (68)	173 (206)	Normal	[34]
CIN2/3 treatment	3662 (4354)	1832 (2178)	5492 (6530)	Normal	[34]
Colposcopy	350 (416)	174 (207)	526 (625)	Normal	[34]
Biopsy	500 (595)	250 (297)	750 (892)	Normal	[34]
Colposcopies with biopsies	8%	-	-	-	^†^
Stage I cancer treatment	28,350 (33,710)	14,176 (16,856)	42,524 (50,564)	-	[34]
Stage II cancer treatment	34,568 (41,103)	17,284 (20,552)	51,852 (61,655)	-	[34]
Stage III cancer treatment	34,568 (41,103)	17,284 (20,552)	51,852 (61,655)	-	[34]
Stage IV cancer line 1 treatment	43,016 (51,149)	21,508 (25,574)	64,524 (76,723)	-	[34]
Stage IV cancer line 2 treatment	75,552 (89,836)	37,776 (44,918)	113,328 (134,754)	-	[34]
Cancer treatment ^‡^	37,227 (44,265)	29,781 (35,412)	44,672 (53,118)	Normal	Calculated
XGT cost factor	1.15	1.00	1.30	-	^†^
**Utility**					
Screening	0.980	0.970	0.990	-	[34]
Colposcopy normal results	0.950	0.924	0.976	-	[34]
CIN1	0.910	0.888	0.954	-	[34]
CIN2/3	0.870	0.804	0.936	-	[34]
Cancer Stage I	0.650	0.490	0.810	-	[34]
Cancer Stage II/III	0.560	0.420	0.700	-	[34]
Cancer Stage IV	0.480	0.360	0.600	-	[34]
Cancer stage I survivor	0.970	0.730	0.990	-	[34]
Cancer stage II/III survivor	0.900	0.680	0.990	-	[34]
Cancer stage IV survivor	0.620	0.470	0.780	-	[34]
**QALY loss**					
Screening	0.000769	0.000384	0.00115	Normal	Calculated
CIN1 or negative for CIN1	0.00538	0.00269	0.00723	Normal	Calculated
CIN2/3	0.0200	0.00985	0.0302	Normal	Calculated
Cancer treatment ^‡^	0.0930	0.0640	0.121	Normal	Calculated
Average lifetime QALY loss for cancer ^‡^	18.7	14.9	22.4	Normal	Calculated

* repeat screening, post-colposcopy for CIN1/negative for CIN; † assumption; ‡ weighted by stage. Abbreviations: ASCUS: atypical squamous cells of undetermined significance; ASC-H: atypical squamous cells cannot exclude high-grade squamous intraepithelial lesion; CIN: cervical intraepithelial neoplasia; hrHPV: human papillomavirus genotypes; HSIL: high-grade squamous intraepithelial lesion; PGT: HPV partial genotyping; NILM: negative for intraepithelial lesion or malignancy; XGT: HPV extended genotyping.

The total cost and QALY loss with XGT were compared to PGT. The difference in costs divided by the difference in QALY loss provided the incremental cost-effective ratio (ICER). All costs and QALY losses were discounted at 3.0% annually, in line with ACE recommendations [57]. The cost-effectiveness threshold was taken as SGD 100,000 (USD 118,906), comparable to the gross domestic product per capita in Singapore in 2021 (SGD 97,798) [58].

The authors apologize for any inconvenience caused and state that the scientific conclusions are unaffected. The original article has been updated.
