# Supplementary material for: Cost-Effectiveness Analysis of HPV Extended versus Partial Genotyping for Cervical Cancer Screening in Singapore
Source: Cancers (Basel). 2023 Mar 16;15(6):1812. doi: 10.3390/cancers15061812 (PMC10046888; doi:10.3390/cancers15061812)
Supplement: Supplementary file 1 [file cancers-15-01812-s001.zip › cancers-2234307-supplementary.pdf]

Table S1: Assumptions for decision model comparing XGT and PGT

S/N	Assumption
**1**	Women attending screening are unvaccinated as the national school-based HPV vaccination program was introduced only in 2019 [1].
**2**	Follow-up of abnormal results with colposcopy, and follow-up post-CIN2/3 treatment is 100%
**3**	Follow-up for repeat screening and for CIN0/1 post-colposcopy is the same (75%)
**4**	Colposcopy is 100% accurate (0% false positive rate, 0% false negative rate)
**5**	Follow-up post-colposcopy<br>- Modelled according to Singapore management guidelines [2], and does not differ by genotype<br>- All ASCUS/LSIL post-colposcopy for no lesion/CIN1 managed as for NILM<br>- CIN1 or negative for CIN are followed for up to 4 years, according to time horizon<br>- All CIN2/3 are treated and return to regular screening with no recurrence as recurrence is low and not expected affect differences significantly. Hence no long-term consequences modelled.
**6**	For cancer:<br>- Quality of life among cancer survivors is similar to without cancer after 10 years of treatment [3].<br>- All diagnosed cancers are treated<br>- A weighted average life years lost, disutility and treatment costs applied to every cancer
**7**	Clinic visit costs are counted for screening, follow-up post treatment, and colposcopy. Visit costs assumed to be accounted for in Cancer and CIN treatment costs
**8**	PGT and XGT are PCR based technologies and 100% accurate in the ability to detect and type HPV virus
**9**	Group B genotypes with ASCUS assumed to have similar risk of CIN2+ at repeat screening as non-HPV16/18 with NILM
**10**	Duration of disutility<br>- Screening: 2 weeks<br>- Colposcopy (negative for CIN): 4 weeks<br>- CIN1 : 4 weeks<br>- CIN2/3: 8 weeks<br>- Cancer stage I: 6 weeks<br>- Cancer stage II/III: 10 weeks<br>- Cancer stage III: 24 weeks<br>- Cancer survivor: 52 weeks

Abbreviations: ASCUS, atypical squamous cells of undetermined significance; CIN, cervical intraepithelial neoplasia; hrHPV, high-risk human papillomavirus; LSIL, low-grade squamous intraepithelial lesion; PCR, polymerase chain reaction; PGT, HPV partial genotyping; NILM, negative for intraepithelial lesion or malignancy; Neg, negative for CIN; XGT, HPV extended genotyping

Scenario analysis

Scenario 1: effect of detecting potentially missed cancers one year later in XGT for Group B genotypes with ASCUS. In analysis 1, potentially missed cancers in XGT were assumed to be detected and screened without any additional cost and QALY loss. In analysis 2, potentially missed cancers in XGT were assumed to be detected and treated with additional 20% cost and QALY loss.

Table S2: Cost and outcomes for scenario 1

	QALY loss	Cost of HPV	Cost of cytology	Cost of colposcopy	Cost of clinic consult	Total cost	ICER
**Base case:** additional cost and QALY = 0%, no detection of possibly missed cancer modelled 1 year after initial screening in XGT							
**PGT**	-6528.3	SG$62,106,581 (US$73,848,491)	SG$5,953,061 (US$7,078,551)	SG$14,259,917 (US$16,955,906)	SG$45,038,254 (US$53,553,215)	SG$145,904,751 (US$173,489,597)	-
**XGT**	-6253.88	SG$71,753,834 (US$85,319,660)	SG$5,658,456 (US$6,728,247)	SG$11,486,606 (US$13,658,271)	SG$44,310,726 (US$52,688,140)	SG$150,396,871 (US$178,831,000)	**SG$16,370/QALY (US$19,465/QALY)**
**Difference**	274.42	SG$9,647,253 (US$11,471,169	- SG$294,605 (- US$350,303)	- SG$2,773,311 (- US$3,297,635)	- SG$727,528 (- US$865,075)	SG$4,492,120 (US$5,341,403)	-
**Analysis 1:** additional cost and QALY = 0%, detection of possibly missed cancer modelled 1 year after initial screening in XGT							
**PGT**	-6528.3	SG$62,106,581 (US$73,848,491)	SG$5,953,061 (US$7,078,551)	SG$14,259,917 (US$16,955,906)	SG$45,038,254 (US$53,553,215)	SG$145,904,751 (US$173,489,597)	-
**XGT**	-6415.45	SG$71,751,619 (US$85,317,026)	SG$5,657,133 (US$6,726,674)	SG$11,486,606 (US$13,658,271)	SG$44,309,470 (US$52,686,647)	SG$150,681,886 (US$179,169,900)	**SG$42,332/QALY US$50,335/QALY**
**Difference**	112.85	SG$9,645,038 (US$11,468,535)	- SG$295,928 (- US$351,876)	- SG$2,773,311 (US$3,297,635)	- SG$728,784 (- US$866,568)	SG$4,777,135 (US$5,680,303)	-
**Analysis 2:** additional cost and QALY = 20%, detection of possibly missed cancer modelled 1 year after initial screening in XGT							
**PGT**	-6528.3	SG$62,106,581 (US$73,848,491)	SG$5,953,061 (US$7,078,551)	SG$14,259,917 (US$16,955,906)	SG$45,038,254 (US$53,553,215)	SG$145,904,751 (US$173,489,597)	-
**XGT**	-6447.89	SG$71,751,619 (US$85,317,026)	SG$5,657,133 (US$6,726,674)	SG$11,486,606 (US$13,658,271)	SG$44,309,470 (US$52,686,647)	SG$150,746,171 (US$179,246,339)	**SG$60,209/ QALY US$71,592/ QALY**
**Difference**	80.41	SG$9,645,038 (US$11,468,535)	- SG$295,928 (- US$351,876)	- SG$2,773,311 (US$3,297,635)	- SG$728,784 (- US$866,568)	SG$4,841,420 (US$5,756,742)	-

Abbreviations: HPV, human papillomavirus; ICER, incremental cost-effectiveness ratio; PGT, HPV partial genotyping; XGT, HPV extended genotyping; QALY, quality-adjusted life years

Table S3: Resource utilization for scenario 1

	Cancers treated	CIN2/3 treated	CIN2+ treated	No. of colposcopy	No of HPV test	No. of cytology	No. of clinic visits
**Base case:** additional cost and QALY = 0%, no detection of possibly missed cancer modelled 1 year after initial screening in XGT							
**PGT**	106	3993	4099	36809	540799	86419	601796
**XGT**	98	3701	3799	29679	543245	80392	592009
**Difference**	-8	-292	-300	-7130	2446	-6027	-9787
**Analysis 1:** additional cost and QALY for = 0%, detection of possibly missed cancer modelled 1 year after initial screening in XGT							
**PGT**	106	3993	4099	36809	540799	86419	601796
**XGT**	107	3692	3799	29679	543228	80375	591992
**Difference**	1	-301	-300	-7130	2429	-6044	-9804
**Analysis 2:** additional cost and QALY for = 20%, detection of possibly missed cancer modelled 1 year after initial screening in XGT							
**PGT**	106	3993	4099	36809	540799	86419	601796
**XGT**	107	3692	3799	29679	543228	80375	591992
**Difference**	1	-301	-300	-7130	2429	-6044	-9804

Abbreviations: CIN, cervical intraepithelial neoplasia; PGT, HPV partial genotyping; XGT, HPV extended genotyping; QALY, quality-adjusted life years

Scenario 2: effect of varying risk of CIN2+ and proportion of cancers among CIN2+ diagnosed in initial screening, among patients with Group B genotypes and ASCUS.
In analysis 1, the upper limit for both CIN2+ risk and proportion of cancers diagnosed among CIN2+ for patients with Group B genotypes with ASCUS was analyzed. In analysis 2, the lower limit for both inputs was analyzed.

Table S4: Cost and outcomes for scenario 2

	QALY loss	Cost of HPV	Cost of Cytology	Cost of Colposcopy	Cost of clinic consult	Total cost	ICER
**Base case:** proportion of cancer among CIN2+ = 2.6%, baseline CIN2+ risk = 6.05%							
**PGT**	-6528.3	SG$62,106,581 (US$73,848,491)	SG$5,953,061 (US$7,078,551)	SG$14,259,917 (US$16,955,906)	SG$45,038,254 (US$53,553,215)	SG$145,904,751 (US$173,489,597)	-
**XGT**	-6253.88	SG$71,753,834 (US$85,319,660)	SG$5,658,456 (US$6,728,247)	SG$11,486,606 (US$13,658,271)	SG$44,310,726 (US$52,688,140)	SG$150,396,871 (US$178,831,000)	**SG$16,370/QALY (US$19,465/QALY)**
**Difference**	274.42	SG$9,647,253 (US$11,471,169	- SG$294,605 (- US$350,303)	- SG$2,773,311 (- US$3,297,635)	- SG$727,528 (- US$865,075)	SG$4,492,120 (US$5,341,403)	-
**Analysis 1:** proportion of cancer among CIN2+ = 10.0%, baseline CIN2+ risk = 7.39%							
**PGT**	-7249.37	SG$62,111,537 (US$73,854,384)	SG$5,953,089 (US$7,078,584)	SG$14,254,033 (US$16,948,036)	SG$45,034,739 (US$53,549,036)	SG$147,517,315 (US$175,407,033)	-
**XGT**	-6500.53	SG$71,748,885 (US$85,313,775)	SG$5,657,209 (US$6,726,765)	SG$11,482,005 (US$13,652,800)	SG$44,306,426 (US$52,683,027)	SG$150,379,412 (US$178,810,240)	**SG$3,822/QALY (US$4,545/QALY)**
**Difference**	748.84	SG$9,637,348 (US$11,459,391)	- SG$295,879 (- US$351,818)	- SG$2,772,027 (- US$3,296,108)	- SG$728,313 (- US$866,008)	SG$2,862,098 (US$3,403,208)	-
**Analysis 2:** proportion of cancer among CIN2+ = 0%, baseline CIN2+ risk = 1.18%							
**PGT**	-6345.05	SG$62,057,099 (US$73,789,654)	SG$5,931,717 (US$7,053,171)	SG$14,281,963 (US$16,982,120)	SG$45,031,259 (US$53,544,898)	SG$144,309,977 (US$171,593,314)	-
**XGT**	-6187.29	SG$71,755,170 (US$85,321,249)	SG$5,658,792 (US$6,728,647)	SG$11,487,848 (US$13,659,748)	SG$44,311,887 (US$52,689,521)	SG$150,401,584 (US$178,836,604)	**SG$38,613/QALY (US$45,913/QALY)**
**Difference**	157.76	SG$9,698,071 US$11,531,595	- SG$272,925 (- US$324,524)	- SG$2,794,115 (- US$3,322,372)	- SG$719,372 (- US$855,377)	SG$6,091,607 (US$7,243,290)	-

Abbreviations: HPV, human papillomavirus; ICER, incremental cost-effectiveness ratio; PGT, HPV partial genotyping; XGT, HPV extended genotyping; QALY, quality-adjusted life years

Table S5: Resource utilization for scenario 2

	Cancers treated	CIN2/3 treated	CIN2+ treated	No. of colposcopy	No of HPV test	No. of cytology	No. of clinic visits
**Base case:** proportion of cancer among CIN2+ = 2.6%, baseline CIN2+ risk = 6.05%							
**PGT**	106	3993	4099	36809	540799	86419	601796
**XGT**	98	3701	3799	29679	543245	80392	592009
**Difference**	-8	-292	-300	-7130	2446	-6027	-9787
**Analysis 1:** proportion of cancer among CIN2+ = 10.0%, baseline CIN2+ risk = 7.39%							
**PGT**	145	4041	4185	36793	540840	86385	601746
**XGT**	98	3700	3798	29667	543207	80373	591951
**Difference**	-47	-341	-387	-7126	2367	-6012	-9795
**Analysis 2:** proportion of cancer among CIN2+ = 0%, baseline CIN2+ risk = 1.18%							
**PGT**	95	3679	3774	36867	540376	86279	601714
**XGT**	98	3701	3799	29682	543255	80398	592025
**Difference**	3	22	25	-7185	2879	-5881	-9689

Abbreviations: CIN, cervical intraepithelial neoplasia; PGT, HPV partial genotyping; XGT, HPV extended genotyping

Scenario 3: Effect of varying HPV burden

In analysis 1, data inputs from Portugal were used for HPV prevalence, proportion of non-HPV16/18 infections and proportion of Group B genotypes among non-HPV16/18 infections [4]. In analysis 2, the lower limit for high-risk HPV prevalence from Portugal was used, as the point estimate used in analysis 1 was higher than the prevalence of high-risk HPV in Singapore.

Table S6: Cost and outcomes for scenario 3

	QALY loss	Cost of HPV	Cost of cytology	Cost of colposcopy	Cost of clinic consult	Total cost	ICER
**Base case:** HPV prevalence= 9.2%, proportion of non-HPV16/18 infections = 80.8%, proportion of group B genotypes among non-HPV16/18 infections = 56.6%							
**PGT**	-6528.3	SG$62,106,581 (US$73,848,491)	SG$5,953,061 (US$7,078,551)	SG$14,259,917 (US$16,955,906)	SG$45,038,254 (US$53,553,215)	SG$145,904,751 (US$173,489,597)	-
**XGT**	-6253.88	SG$71,753,834 (US$85,319,660)	SG$5,658,456 (US$6,728,247)	SG$11,486,606 (US$13,658,271)	SG$44,310,726 (US$52,688,140)	SG$150,396,871 (US$178,831,000)	**SG$16,370/QALY (US$19,465/QALY)**
**Difference**	274.42	SG$9,647,253 (US$11,471,169	- SG$294,605 (- US$350,303)	- SG$2,773,311 (- US$3,297,635)	- SG$727,528 (- US$865,075)	SG$4,492,120 (US$5,341,403)	-
**Analysis 1:** HPV prevalence= 10.2%, proportion of non-HPV16/18 infections = 83.3%, proportion of group B genotypes among non-HPV16/18 infections = 62.5%							
**PGT**	-7058.69	SG$62,589,925 (US$74.423.216)	SG$6,639,725 (US$7,895,036)	SG$15,605,141 (US$18,555,459)	SG$45,792,509 (US$54,450,070)	SG$149,969,517 (US$178,322,850)	-
**XGT**	-6710.91	SG$72,392,918 (US$86,079,570)	SG$6,257,765 (US$7,440,862)	SG$12,121,678 (US$14,413,410)	SG$44,878,237 (US$53,362,945)	SG$153,250,801 (US$182,224,496)	**SG$9,435/QALY US$11,219/QALY**
**Difference**	347.78	SG$9,802,993 (US$11,656,353)	- SG$381,961 (US$454,175)	- SG$3,483,463 (- US$4,142,049)	- SG$914,273 (- US$1,087,126)	SG$3,281,284 (US$3,901,646)	-
**Analysis 2:** HPV prevalence= 7.9%, proportion of non-HPV16/18 infections = 83.3%, proportion of group B genotypes among non-HPV16/18 infections = 62.5%							
**PGT**	-5553.78	SG$61,445,366 (US$73,062,266)	SG$5,142,532 (US$6,114,782)	SG$12,086,335 (US$14,371,385)	SG$43,924,697 (US$52,229,128)	SG$137,579,667 (US$163,590,567)	-
**XGT**	-5284.41	SG$70,983,209 (US$84,403,340)	SG$4,846,700 (US$5,763,020)	SG$9,388,358 (US$11,163,327)	SG$43,216,584 (US$51,387,139)	SG$142,066,382 (US$168,925,543)	**SG$16,656/QALY US$19,805/QALY**
**Difference**	269.37	SG$9,537,843 (US$11,341,074)	- SG$295,832 (- US$351,762)	- SG$2,697,977 (- US$3,208,058)	- SG$708,113 (- US$841,989)	SG$4,486,715 (US$5,334,976)	-

Abbreviations: HPV, human papillomavirus; ICER, incremental cost-effectiveness ratio; PGT, HPV partial genotyping; XGT, HPV extended genotyping; QALY, quality-adjusted life years

Table S7: Resource utilization for scenario 3

	Cancers treated	CIN2/3 treated	CIN2+ treated	No. of colposcopy	No of HPV test	No. of cytology	No. of clinic visits
**Base case:** additional cost and QALY for = 0%, no detection of possibly missed cancer modelled 1 year after initial screening in XGT							
**PGT**	106	3993	4099	36809	540799	86419	601796
**XGT**	98	3701	3799	29679	543245	80392	592009
**Difference**	-8	-292	-300	-7130	2446	-6027	-9787
**Analysis 1:** additional cost and QALY for = 0%, detection of possibly missed cancer modelled 1 year after initial screening in XGT							
**PGT**	110	4164	4275	40283	545080	96224	611993
**XGT**	100	3790	3891	31327	548140	88505	599695
**Difference**	-10	-374	-384	-8956	3060	-7719	-12298
**Analysis 2:** additional cost and QALY for = 20%, detection of possibly missed cancer modelled 1 year after initial screening in XGT							
**PGT**	86	3225	3311	31200	534942	74527	586768
**XGT**	78	2936	3013	24263	537312	68548	577242
**Difference**	-8	-289	-298	-6937	2370	-5979	-9526

Abbreviations: CIN, cervical intraepithelial neoplasia; PGT, HPV partial genotyping; XGT, HPV extended genotyping; QALY, quality-adjusted life years

References

1. Ministry of Health. HPV school-based programme. Availabe online: https://www.moh.gov.sg/news-highlights/details/hpv-school-based-programme (accessed on 3 January 2023).
2. The Society for Colposcopy & Cervical Pathology of Singapore. Management guidelines for cervical screening & preinvasive disease of the cervix. . Availabe online: https://www.sccps.org/wp-content/uploads/2019/03/CSS-Clinical-Mgt-Guidelines-2019_March-Release.pdf (accessed on 20 December 2022).
3. Arndt, V.; Koch-Gallenkamp, L.; Jansen, L.; Bertram, H.; Eberle, A.; Holleczek, B.; Schmid-Höpfner, S.; Waldmann, A.; Zeissig, S.R.; Brenner, H. Quality of life in long-term and very long-term cancer survivors versus population controls in Germany. *Acta Oncol* **2017**, *56*, 190-197, doi:10.1080/0284186x.2016.1266089.
4. Sousa, H.; Tavares, A.; Campos, C.; Marinho-Dias, J.; Brito, M.; Medeiros, R.; Baldaque, I.; Lobo, C.; Leça, L.; Monteiro, P., et al. High-Risk human papillomavirus genotype distribution in the Northern region of Portugal: Data from regional cervical cancer screening program. *Papillomavirus Res* **2019**, *8*, 100179, doi:10.1016/j.pvr.2019.100179.
